# Supplementary material for: The genome sequence of the Diamondback Moth, Plutella xylostella (Linnaeus, 1758)
Source: Wellcome Open Res. 2023 Sep 18;8:404. [Version 1] doi: 10.12688/wellcomeopenres.20006.1 (PMC11109710; doi:10.12688/wellcomeopenres.20006.1)
Supplement: Peer review history

# Reviewer report: Reviewer response for version 1

**DOI:** 10.21956/wellcomeopenres.22152.r78120

**Published:** 2024-05-10

**Authors:** Muzafar Riyaz

**Affiliations:** 1 Xavier Research FoundationSt. Xavier's College, Palayamkottai, Tamil Nadu, India

The paper presents a comprehensive account of the genomic sequencing and annotation of the Diamondback Moth, Plutella xylostella (Linnaeus, 1758), a significant agricultural pest. Through detailed descriptions of sample collection, sequencing methodologies, assembly techniques, and annotation processes, the study achieves a high-quality genome assembly with chromosome-level scaffolding and accurate gene annotations. The background section provides context on the pest's agricultural significance, referencing previous molecular studies, while the methods section outlines experimental procedures and software tools utilized, ensuring reproducibility and transparency. Figures and tables have aided in visualizing assembly metrics, and the data availability statement provides accession numbers for raw sequence data and the genome assembly, facilitating accessibility for further research. The extensive list of references demonstrates a thorough review of relevant literature, supporting the study's findings within the broader scientific context. Overall, this paper contributes valuable genomic resources for P. xylostella research, aiding in pest management strategies and advancing understanding in insect genomics.

Are sufficient details of methods and materials provided to allow replication by others?

Yes

Is the rationale for creating the dataset(s) clearly described?

Yes

Are the datasets clearly presented in a useable and accessible format?

Yes

Are the protocols appropriate and is the work technically sound?

Yes

Reviewer Expertise:

Insect genomics, Moths, Phylogenetics

I confirm that I have read this submission and believe that I have an appropriate level of expertise to confirm that it is of an acceptable scientific standard.

## References

[1] : The genome sequence of the Diamondback Moth, Plutella xylostella. *researchgate.net* .2023;10.12688/wellcomeopenres.20006.1PMC1110971038779055

# Reviewer report: Reviewer response for version 1

**DOI:** 10.21956/wellcomeopenres.22152.r82027

**Published:** 2024-05-09

**Authors:** Kyle M Benowitz

**Affiliations:** 1 Austin Peay State University (Ringgold ID: 2536), Clarksville, Tennessee, USA

The paper reports a new chromosome-level genome assembly for the lepidopteran pest *Plutella xylostella*. The assembly and annotation are of high quality, and the genome should be useful for other researchers in the field. The methods are current and easy to follow. Overall, the paper is well written, and I just have a few minor comments.

 Minor comments: 

 1. There's a small confusion in the methods, as it is stated in the 2nd paragraph of the methods that tissue from ilPluXylo3 was 'set aside for Hi-C sequencing'. However, in all other parts of the report it is made clear that tissue from a separate individual, ilPluXylo4, was used for Hi-C.

 2. In the methods for genome annotation, what transcriptomic data was used? What tissues/life stages were it from, and where can that data be found? This is important for the replicability of the work.

 3. Given that a chromosome-level assembly for this species already exists, a brief sentence comparing the two assemblies could be added. Since this paper is just a data note, a large amount of comparative analysis or interpretation would be inappropriate; however, a quick statement noting the similarity in size/structure would be useful for readers.

Are sufficient details of methods and materials provided to allow replication by others?

Partly

Is the rationale for creating the dataset(s) clearly described?

Yes

Are the datasets clearly presented in a useable and accessible format?

Yes

Are the protocols appropriate and is the work technically sound?

Yes

Reviewer Expertise:

I work in the field of insect evolutionary genomics. I have been involved with several genome sequencing projects, including that of the lepidopteran pest Helicoverpa zea.

I confirm that I have read this submission and believe that I have an appropriate level of expertise to confirm that it is of an acceptable scientific standard.

# Reviewer report: Reviewer response for version 1

**DOI:** 10.21956/wellcomeopenres.22152.r80234

**Published:** 2024-05-08

**Authors:** Jerome H L Hui

**Affiliations:** 1 The Chinese University of Hong Kong, Hong Kong, Hong Kong

Boyes and colleagues report the genome sequence of a male diamondblack/cabbage moth *Plutella xylostella *(Linnaeus, 1758). As one can judge from its common name, it is an important pest of brassicaceous crops such as cabbage. This species is well known for its migratory tendencies and now become a cosmopolitan species. In Britain, the immediate sources of this species have been suggested to be coming from countries in the western part of continental Europe (Wainwright et al 2020 Insects). As the authors have pointed out, certain level of molecular data of this species have already been obtained previously, including transcriptomes and draft genomes (N50 = 2.2 kb and 737kb from two studies, Jouraku et al 2013 BMC Genomics [Ref 2]; You et al 2013 Nature Genetics [Ref 3]). Nevertheless, it is necessary to point out that this report contains a much improved high-quality genome resource. It will be useful for a range of further studies, including from not limited to, revealing their population structures, dissecting host plant-insect relationships, to understanding their evolution with other lepidopterans, which have important values in both basic knowledge and applications.

 This genome resource is excellent from the summary statistics, with high BUSCO numbers, high sequence continuity (scaffold N50), and majority of sequences contained on the 32 pseudochromosomes (plus mitochondrion). All in all, this is another valuable contribution by the Darwin Tree of Life Consortium.

Are sufficient details of methods and materials provided to allow replication by others?

Yes

Is the rationale for creating the dataset(s) clearly described?

Yes

Are the datasets clearly presented in a useable and accessible format?

Yes

Are the protocols appropriate and is the work technically sound?

Yes

Reviewer Expertise:

Genomics, evolution, invertebrates

I confirm that I have read this submission and believe that I have an appropriate level of expertise to confirm that it is of an acceptable scientific standard.

## References

[1] : KONAGAbase: a genomic and transcriptomic database for the diamondback moth, Plutella xylostella. *BMC Genomics* .2013;14: 10.1186/1471-2164-14-464 464 10.1186/1471-2164-14-464 23837716 PMC3711893

[2] : A heterozygous moth genome provides insights into herbivory and detoxification. *Nat Genet* .2013;45(2) : 10.1038/ng.2524 220-5 10.1038/ng.2524 23313953
